# Use of hollow fiber systems to model treatment of infections: A practical guide

**DOI:** 10.1016/j.isci.2026.115535

**Published:** 2026-03-31

**Authors:** Jelmer Raaijmakers, Fernando Sanz-García, Zahra Sadouki, Diana A. Aguilar-Ayala, Andrew Mead, John B. Readman, Timothy D. McHugh, L. Pelligand, Joseph F. Standing, Santiago Ramón-García, Jakko van Ingen, Frank Kloprogge, Ainhoa Lucía, the European Society of Clinical Microbiology and Infectious Diseases (ESCMID) PK/PD of Anti-Infectives Study Group (EPASG)

**Affiliations:** 1 Radboud Centre for Infectious Diseases, Department of Medical Microbiology, Radboud University Medical Centre, Nijmegen, the Netherlands; 2 Department of Microbiology, Paediatrics, Radiology and Public Health, Faculty of Medicine, University of Zaragoza, Zaragoza, Spain; 3 UCL Centre for Clinical Microbiology, Division of Infection & Immunity, University College London, London, UK; 4 Department of Pathobiology and Population Sciences, APPRAISE Lab, The Royal Veterinary College, London, UK; 5 Infection, Immunity and Inflammation Department, Great Ormond Street Institute of Child Health, University College London, London, UK; 6 Department of Pharmacy, Great Ormond Street Hospital for Children, London, UK; 7 Research & Development Agency of Aragón Foundation (Fundación ARAID), Zaragoza, Spain; 8 Spanish Network for Research on Respiratory Diseases (CIBERES), Carlos III Health Institute, Madrid, Spain; 9 Institute for Global Health, University College London, London, UK

**Keywords:** natural sciences, biological sciences, microbiology, medical microbiology

## Abstract

This EPASG-endorsed consensus report provides practical and standardized guidance for the use of hollow fiber systems (HFSs) in modeling antimicrobial treatment of bacterial infections. An HFS allows the simulation of dynamic pharmacokinetic (PK) profiles and bacterial responses over extended time periods, making it highly suited for PK/pharmacodynamic (PD) studies. This document serves as a foundational reference for researchers using HFS to evaluate antimicrobial efficacy, optimize dosing regimens, and support translational research in infectious diseases.

## Scope of the document

### Overview

This document describes best practice in the conduct of experiments with hollow fiber systems (HFSs) and can be used as a guide for setting up these experiments in studies of intracellular or extracellular bacterial infection. In line with the recent EMA (European Medicines Agency) guidance,^1^ we have adopted the term “hollow fiber system” (HFS), rather than “hollow fiber infection model” (HFIM) or “hollow fiber model” (HFM), where “hollow fiber” refers to a hollow cylinder with semi-permeable membrane “fibers” running through. Using this terminology, the specific infection can be identified using HFS-I (infection), for example, HFS-TB for an HFS for tuberculosis.

Historically, antibiotic research has predominantly relied on murine models, such as thigh and lung infection models, to bridge the gap between bench and bedside.^2^ These models facilitate the study of drug-induced toxicity, bacterial load, and the emergence of drug resistance over time. However, as only small and limited numbers of samples can be obtained in such experiments, detailed investigation of bacterial responses remains constrained. The HFS overcomes several of these limitations by allowing larger and more frequent sampling from the bacterial population, thereby enabling more comprehensive analyses of bacterial responses to antibiotic therapy. In addition, the use of animals inherently raises ethical considerations. A further limitation of murine models lies in the pharmacokinetic (PK) discrepancies between animals and humans, which can compromise the translational relevance of the findings.^3^ The HFS addresses these challenges by simulating human or animal PK, while reducing the need for animal experimentation.

This document first outlines a brief overview of the experimental setup of the HFS-I, followed by the requirements for performance of hollow fiber experiments in terms of equipment, laboratory layout, technical setup, preparation, and practical execution. The document also refers to minimum reporting standards to ensure reproducibility of the experiments.

### HFS for bacterial studies

**Figure 1:**
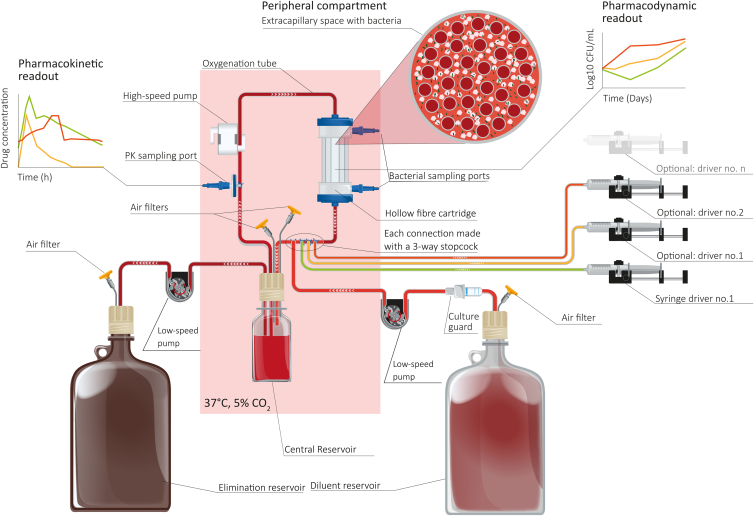
Schematic overview of the HFS This system is defined as the complete set of diluent reservoir, central reservoir, hollow fiber cartridge, elimination reservoir, the pumps, and tubing.

An HFS can be set up in various ways; here, we describe a commonly used and robust approach (Figure 1). This experimental setup has been shown to be efficient for the study of aerobic bacterial responses to antimicrobial therapy such as, but not limited to, defining pharmacodynamic (PD) drivers and testing different posology to inform clinical trials and evaluate drug combination effects.^4^ The flexibility of the experimental setup provides an *in vitro* tool for simulation of dynamic *in vivo* antimicrobial concentration-time profiles, e.g., those occurring at the site of infection (Appendix B; Figure 4).^1^

The HFS uses hollow fiber cartridges, which are closed cylinders with semi-permeable fibers running through them forming a closed loop system when connected to inflow and outflow tubes to a central reservoir.^1^^,^^4^ PK profiles, i.e., absorption, distribution, and metabolism/elimination, can be simulated in the HFS by administering drug(s) in the central reservoir containing growth medium and manipulating the drug concentrations using pump-controlled syringes and peristaltic pumps. The content of the central reservoir (containing varying concentrations of antibiotics) is pumped through the hollow fibers in the cartridge^1^ and delivered to the bacteria in the extracapillary space (ECS). The hollow fibers are semi-permeable, which allows the exchange of drugs, growth medium, and waste products between the ECS and the intracapillary space (ICS). The cartridge contains the bacterial cells within the enclosed ECS, and the cells can be collected via the sampling ports.^1^ The system enables the cultivation of bacteria and host cells (e.g., monocytes) at high cell densities and can support, depending on the microorganism and cartridge type used, up to ca. 10^12^ bacterial cells/mL for extended periods of time.^5^ This is particularly important for PK/PD studies aimed at investigating the bacterial response to antimicrobial treatment and the capacity to prevent the development of bacterial resistance.

## Considerations prior to setting up HFS

### Laboratory lay-out and requirements

As per ISO (International Organization for Standardization) regulation,^6^ a biosafety containment level 2 or higher is required to ensure the safety of staff and the environment when pathogenic microorganisms are being studied, including with the HFS.

A temperature-controlled incubator, or hot room, is necessary to ensure that the hollow fiber experiment maintains optimal growth conditions for the microorganism of interest. The incubator should be selected or adapted so that the tubing for dilution and elimination can be routed to and from the reservoirs without physical obstruction, and there is sufficient space nearby for the diluent and syringe drivers. A typical setup with two or three HFSs occupies approximately one square meter outside of the incubator, with the central reservoirs and cartridges placed in the incubator. The equipment should be located near electrical outlets to minimize electric cable hazards, with enough power sockets available for all of the pumps. It is advisable to implement alternative emergency electrical systems, such as an uninterruptible power supply, in case there is electric disruption of the main network during an experiment, which could compromise the experimental setup. The diluent and elimination reservoirs can be located outside the incubator, but the central reservoir, hollow fiber cartridge, and circular connecting tubing should be inside an incubator to maintain the desired temperature for cellular growth.

### Equipment

In addition to the hollow fiber cartridges (see hollow fiber cartridges for more information) and a suitable incubator into which the tubes fit, several pumps are needed to perform hollow fiber experiments. High-speed peristaltic pumps circulate fluid between the central reservoir and the cartridge, and low-speed pumps introduce diluent and remove waste from the central reservoir. Automated syringe drivers may also be needed to add drugs into the system for replication of PK profiles other than intravenous bolus administrations. A complete list of equipment and reusable equipment is listed in Appendix D.

#### Hollow fiber cartridges

Hollow fiber cartridges, like dialysis cartridges, are single-use consumables available in different materials, pore sizes, and volumes. The three most used materials for cartridge fibers are polysulfone, cellulose, and polyvinylidene fluoride (PVDF). Fiber materials can be more hydrophilic or hydrophobic but must ensure good culture performance, and each fiber type has its own properties in terms of binding drugs, protein matrices, antibodies, and cytokines. The preferred material of the cartridge fibers should be tested before starting the experiments to determine the suitability of the cartridge for the combination of pathogen and antimicrobial. Cartridge suitability is assessed by performing drug-free growth control curves with the microorganism of interest and evaluating drug binding in the system^7^ in the absence of bacteria. For the latter, drug dispersion through the fibers into the ECS should also be investigated (see validation of antibiotic profiles in the system).

The fiber pore size should be considered to ensure that all nutrients can penetrate the fiber membrane, while the bacterial and cellular cultures remain seeded in the ECS. The volume of the hollow fiber cartridge is crucial depending on the type of experiment. If a high volume of bacteria need to be harvested (e.g., for RNA-seq), cartridges with a higher ECS volume, over 30 mL, may be preferred. As optimal bacterial growth has strict aerobic/anaerobic (O_2_/CO_2_) requirements, the tubing connecting the CR to the hollow fiber cartridge (known as the oxygenation tubing) needs to be suitably gas permeable. As the surface area-to-volume ratio impacts gas exchange, the length of this tubing needs to be adjusted accordingly. The surface area-to-volume ratio (about 450–4000 cm^2^) between the ICS and ECS is also critical as it defines the rate of nutrient (and drug) equilibration, which can impact bacterial growth dynamics and is positively correlated with the number of fibers packed and the corresponding number of pores.

#### High- and low-speed peristaltic pumps

**Figure 2:**
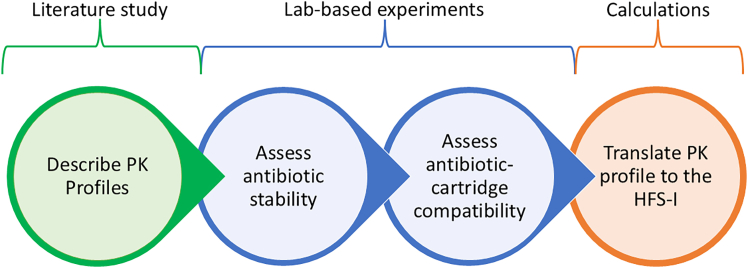
A red-light/green-light approach can be applied to validate the antibiotic profiles within the system Briefly, the process begins with defining the PK profiles to be simulated, based on a comprehensive literature review. Next, the stability of the antibiotic and its compatibility with the cartridge are assessed (for details, see Appendices A and B). Finally, parameters for fresh medium inflow/outflow and drug inflow are calculated, using Appendix C or freely available applications such as HF-App^13^ or PKPDIA.^14^

A (peristaltic) pump provides a constant circular flow through the cartridge, to and from the central reservoir. To achieve equilibrium of nutrients and drugs between the hollow fiber cartridge and the central reservoir, a peristaltic pump is required that can reach medium-to-high speeds. The flow rate should be adjusted so that one-third of the total system volume is circulated each minute. The total system volume includes the central reservoir, the tubing to and from the cartridge, and the cartridge volume.^8^ This ensures that the recirculation-to-feed flow rate ratio remains greater than 10, thereby maintaining an even distribution of an antibiotic throughout the system. The dilution of a drug in the central reservoir is achieved by a low-speed peristaltic pump feeding diluent into the central reservoir from the diluent reservoir and eliminating waste to the elimination reservoir. Flow rates that ensure simulation of PK elimination half-lives are usually low, even when larger volumes are used, so a peristaltic pump with lower speeds (0.2–5 mL/min) is required. Pumps must be carefully calibrated according to the manufacturer’s instructions. It is advisable to test the chosen flow rates by dispensing large volumes (e.g., 1 L) to corroborate whether pumps are well calibrated before each experiment. When using different pump-head models, each should be calibrated to ensure comparability between the models. A constant volume in the central reservoir is maintained by setting higher flow rates of the peristaltic pump from the central reservoir to the elimination reservoir, or using a higher bore size of tubing for the removal of waste compared to feeding in diluent when the height of the outflow is fixed (see Figure 2 in Central Reservoir Volume).

#### Syringe driver

For an experiment where the drug is infused over a period of time or needs repeated administration, or a combination of both, a programmable syringe driver with programming for multistep infusion or gradient is required. This syringe driver will pump the antibiotics into the central reservoir at the desired rate to simulate the T_max_ of the antibiotic(s) being tested. After reaching T_max_, the pump should remain paused for the rest of the dosing interval in experiments simulating multiple doses. The syringe driver must be calibrated according to the manufacturer’s instructions, and only compatible syringe brands should be used, as barrel internal diameters vary between brands. When setting up the HFS, drug syringes can be connected to the central reservoir by using two different approaches. The first approach is to connect the drug inflow to the tubing leading from the hollow fiber cartridge back into the central reservoir by using a 3-way stopcock. The second approach is to connect the syringe to the tubing leading into the central reservoir from the diluent bottle.

#### Caps and tubing

Caps and tubing should be autoclavable to ensure sterility. Each reservoir should be closed with a tight-fitting cap that is vented. Two ports are required for the diluent and elimination reservoirs: one port connected to the central reservoir, and one port that serves as a vent to prevent a vacuum or excess pressure from developing within the reservoir. The central reservoir cap should incorporate five ports. These include one connection to the diluent reservoir, one to the elimination reservoir, one vent port to safeguard against overfilling or emptying in case of pump malfunction, and two ports connecting the reservoir to the hollow fiber cartridge (Figure 1).

The ([platinum cured] silicone) tubing and the connections between the tubing and the reservoir caps should be long enough to prevent stretching of the tubing and preferably use a tight seal, such as a Luer-lock connection, to prevent leakage. The bore size should match the Luer-lock connections at both ends of the tubing. It is advisable to keep a spare set of sterile tubing to hand in case the tubing used becomes damaged or leaks. Also, the part of the tubing that enters the peristaltic pump flattens out over time, changing the set flow rate and increasing the risk of damage. This section can be replaced when needed, and, therefore, it is advisable to also have extra sterile tubing sections for this usage.

#### Reservoirs

Reservoirs should be sterile, autoclavable, and matched to the maximum volume of each compartment (e.g., the diluent volume equals the elimination volume). At least three reservoirs are needed for each experimental setup including setups without drugs, normally included as controls. Independent of cartridge size, each setup needs at least one central reservoir (150–450 mL), one diluent reservoir (>1 L), and one elimination reservoir (>1 L).

#### Sterilizing the equipment

All consumables (clave connectors, air filters, stopcocks, syringes, etc.) should be purchased sterile and handled in an aseptic manner. All autoclavable equipment and liquid media (phosphate buffered saline; PBS, broth) should be sterilized in an autoclave at 121°C for 15 min before each hollow fiber experiment and handled aseptically. Connection points such as the outer ends of tubing, the ports on the caps of the diluent, and the elimination and central reservoir should be wrapped with aluminum foil before autoclaving the materials. Before connecting tubing and consumables, the aluminum foil is removed and connected immediately to prevent contamination of the system (ideally in a Class 2 biological safety cabinet). It is often useful to make as many connections as possible prior to autoclaving to minimize risk at setup. Tubes and fittings can also be capped (e.g., with the Bio-Rad low-pressure systems fitting kit no.731-8220).

### Validation of antibiotic profiles in the system

#### Describing PK profiles

Before starting an HFS experiment, the investigator should determine the desired C_max_, T_max_, and T_1/2_ of the study drug(s), e.g., at the site of infection (blood or specific tissue) (Figure 2), from *in vivo* studies, the literature, or any other proprietary source (Figure 2). It should be recognized that only the free drug fraction is considered active, and, therefore, *in vivo* protein binding should be taken into consideration. However, depending on the scientific question to be answered, other assumptions may be considered more relevant. Nevertheless, it is important to report what has been simulated to allow replication.^9^ To guide the reader in understanding the target PK exposure and its experimental attainment, a visual plot can strengthen the results section. Such plots may, for example, overlay simulated concentrations with the target exposure to aid interpretation or highlight differences between the target and achieved concentrations.

#### Antibiotic stability

The antibiotic solution(s) should be prepared according to the manufacturer’s recommendations and adjusted for purity/potency if a non-formulated drug is being used. The concentration of the drug in solution may decrease over time due to photosensitivity, thermostability, and interactions with the solvent and diluents, which must be taken into account. Loss or degradation in the assay media and of the stock solutions, if they are to be stored for long periods, should be tested to quantify drug degradations kinetics outside the HFS; this also applies to samples taken from the HFS, which could degrade upon storage at −80°C until quantification. Freshly prepared solutions are recommended for every experiment. Loss or degradation of antibiotics, when measured, can be accounted for by compensating through an equivalent increase in dosing for the amount that had degraded. When a drug interacts with solvents or diluents, this should also be measured and accounted for, so that the desired drug concentration is reached.

#### Drug compatibility to the system

**Figure 3:**
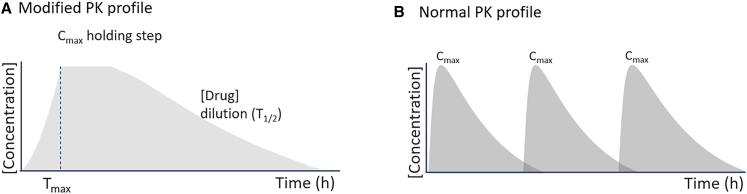
Tests for drug-system compatibility

Not all drugs are compatible with the materials used in the hollow fiber cartridge (e.g., they may not be able to penetrate the semi-permeable membrane or they may bind to the fibers, plastics, or other components of the system, which render them unsuitable to be used together).^10^ The compatibility of the drug(s) of interest with the materials of the system must be assessed prior to conducting an HFS experiment (see Appendix B for detailed information). In this assessment, for the first part, a known concentration of the drug is infused into the system and kept static (i.e., it is not diluted in the system) for ca. 2 h before the drug is diluted. Samples are taken from both the central reservoir and ECS to evaluate whether the drug binds to the fibers and other parts of the HFS, and whether the drug can cross the membranes (Figure 3). In the second part of the assessment, simulation of the desired PK profile is repeated. Samples are taken from both the central reservoir and the ECS to determine the PK within the system, and the antibiotic concentrations should match across these compartments. This type of experiment is necessary to validate the compatibility of the drug with the hollow fiber cartridge and other materials of the system, by establishing whether the drug can penetrate the ECS and equilibrate. If the measurements between the compartments differ (e.g., the drug concentration is lower in the ECS than in the central reservoir), or much lower values than calculated are observed (e.g., due to non-specific binding to the cartridge or plastics), the use of a cartridge with a different fiber type should be considered. This validation should be performed for each drug before conducting a PK/PD experiment. Depending on the frequency of the PK sampling, an equilibrium setting can be observed and maintained. In addition, binding to plastics and/or degradation of drugs in the system can be observed during this experiment. If an antibiotic is found to bind to plastics of the HFS, the tubing between the syringe driver and the HFS should be primed with the antibiotic solution. If proteins are used in the broth in high enough concentrations to be relevant to drug binding to proteins, it may be of interest to measure both free and total drug concentrations. Preliminary experiments outside the HFS can inform on the degree of drug-protein binding in the growth medium of interest.

#### Translation of PK profiles to the HFS

The translation of primary PK parameters (C_max_ or C_max,ss_, T_max_, and T_1/2_) into HFS pump rates can be done using PK equations (see Appendix C), and adding a second central reservoir can allow the simulation of drugs with bi-phasic half-lives.^11^^,^^12^ Freely available software such as HF-App^13^ or PKPDIA^14^ can also be helpful. Note that different freely available software may have different calculation approaches (e.g., whether the low-speed peristaltic pump is on during drug delivery).

Effective flow rates depend on both the bore size of the tubing and the pump rates. Therefore, care should be taken to select the correct bore size to match the pump settings and calibrate the pumps accurately before use, especially if using a low-speed peristaltic pump that expresses rates in volume/time.

## Considerations during the setup of HFS experiments

### Priming the system

#### Pre-washing

Before starting an HFS experiment, it should be checked whether a preservative, such as glycerol, is present in the cartridge. This preservative preserves the fibers by preventing the cartridge from drying out, but it must be washed off before inoculating with bacteria. If it is present, the cartridge and ECS should be washed with pure water, PBS, or the broth used in the experiment. The chances of interaction between the preservative and the washing solution are lower when pure water is used. Only a 1 L central “washing” reservoir containing water/PBS/broth and a two-way cap is used for the washing step. The 1 L “washing” central reservoir is changed to the system central reservoir, with the desired volume for the experiment after the media priming step (see preventing air bubbles in the cartridge). The closed loop system is then mounted on a high-speed peristaltic pump over a 24-h period. At this stage, the setup can be stored before progressing to preventing air bubbles in the cartridge Media priming.

#### Media priming

After the first priming step, the fibers and the system should be saturated with broth, if not already, used in the previous step (pre-washing step). As with washing, this step uses a central “priming” reservoir containing only 1 L of the chosen broth medium and a two-way cap to form a closed circuit. The liquid in the ECS is also replaced with the chosen broth medium by using Luer-lock syringes via the withdrawal ports. The closed loop system is then mounted on a high-speed peristaltic pump over a 24-h period. At this stage, the setup can be stored before progressing to the experiment.

### Maintenance during experiment

#### Central reservoir volume

**Figure 4:**
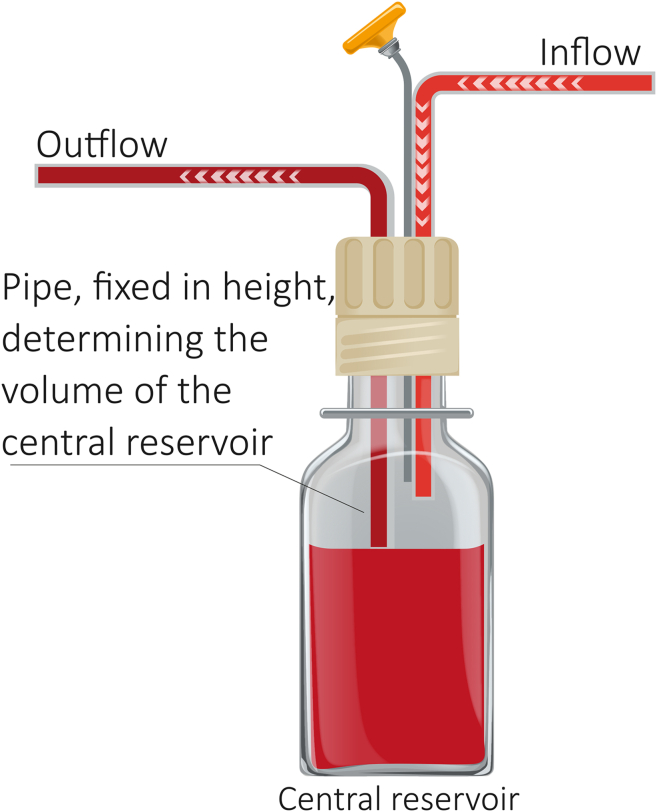
Setting up the volume of the central reservoir

The tubing that drains the liquid from the central reservoir to the elimination reservoir (Figure 4) should be set at a height that corresponds to the desired volume of the central reservoir, thus ensuring that the central reservoir is kept at the chosen constant volume. It is preferable to set the elimination flow rate (CR to elimination reservoir) higher than the diluent flow rate (diluent reservoir to CR) to reduce the risk of “over-filling” the CR, which should retain a constant volume. This can be achieved by using different flow rates for the peristaltic pumps. If independent control of the pump flow rates is not possible, wider-bore tubing from the central reservoir to the elimination reservoir than that from the diluent reservoir to the central reservoir should be used.

The T_1/2_ of the drug and the volume of the central reservoir determine the flow rate between diluent and the central and elimination reservoirs. The volume in the central reservoir can be decreased when setting up the HFS, thereby lowering the inflow rates and thus the volume consumed by the system. Decreasing the volume in the central reservoir tends to decrease the necessary inflow of drug and diluent but increase the relative pump error of the diluent pump. It should be kept in mind that the total volume of this compartment is the sum of the central reservoir, tubing from and to the HFS cartridge, and the ICS and ECS of the cartridge.

#### Preventing air bubbles in the cartridge

To prevent air from accumulating in the hollow fiber cartridge, the central reservoir can be raised to the level of the top of the cartridge throughout the experiment, and a tray with sterile water can be placed in the incubator to increase the environmental humidity. We also recommend manually mixing the contents of the hollow fiber cartridge daily, or at least every other day, and removing air bubbles with syringes connected to the openings of the cartridge.

#### Sterilizing the elimination reservoir

A disinfectant (e.g., Tristel from Tristel Solutions Ltd., Snailwell, UK; or Terralin from Schülke & Mayr, Vienna, Austria) may be added to the elimination reservoir to prevent contamination from growing when a non-sterile elimination reservoir is used.

### Biological considerations of inoculation

#### Bacterial inoculum

Prior to inoculation of the ECS of the hollow fiber cartridges, the bacterial population can be cultured in the preferred medium (typically this is in line with the optimal growth medium prescribed for susceptibility testing as described in ISO 20776-1)^15^ and given sufficient time to reach the logarithmic phase or stationary phase of growth, as desired. Only aerobic bacteria can be used in the HFS-I, as oxygen is inevitably introduced into the medium during circulation. It is well known that the bacterial concentration of the inoculum affects the results of antimicrobial tests.^16^ Therefore, the gold standard of the microdilution inoculum size of ca. 5 ***×*** 10^5^ colony-forming units per milliliter (CFUs/mL) can be used as the starting point. However, other inoculum sizes can be used depending on the research question to be investigated.^7^^,^^17^ In line with good microbiological practice, care should be taken to standardize the bacterial inoculum in terms of storage, passage number, and recovery conditions prior to its use in the HFS.

#### Biofilms and clumping

When experimenting with bacteria that are known to clump (e.g., mycobacteria and nocardiae), detergents (e.g., Tween/Tyloxapol) can be added to the broth. When adding them, it should be kept in mind that they can impact the antibiotic effect, as well as the growth kinetics of the bacteria, because they can alter the structure of the cell wall of the bacteria. Moreover, because biofilms form on the fibers, the contents of the cartridge should be thoroughly mixed through the sampling ports before each sampling by pushing and pulling the contents of the cartridge using syringes.

#### Macrophage models of infection

When a macrophage model of infection is applied to the HFS, the model cells must be able to multiply and not adhere to the fibers and/or other plastics of the cartridge. The duration of the infection of the model cells by the tested bacteria depends on the doubling time of the bacteria. This infection phase can be performed by adding the desired bacterial CFU/mL to the model cell suspension before inoculation of the hollow fiber cartridge. For experiments in which only intracellular bacteria are inoculated into the HFS, the suspension can be centrifuged to separate the infected cells from the extracellular bacteria.

#### Time between cartridge inoculation and the start of the experiment

In the case of rapidly growing bacteria (with generation times of less than an hour), the time between inoculation of the ECS and the first antibiotic administration may alter the bacterial population to the extent that the bacterial density when the first antibiotic dose is administered is no longer than the intended starting density. Therefore, it is important to take a bacterial culture sample just before the first antibiotic administration (T = 0). This is especially important for intracellular infection models, as there is a large decrease in the macrophage cells during inoculation into the HFS. Depending on the research question, it may be desirable to bypass the lag phase. To minimize growth retardation caused by transferring bacteria (and model macrophages) into the HFS, cartridge inoculation should be done at least one bacterial doubling time before administering the first antibiotic dose. The exact timing may vary depending on the pathogen and strain. These observations might not be as relevant in the case of slow growing bacteria, as timing can be controlled and used to modulate the question to be answered by the HFS experiment.^7^

#### Drug-free growth control

In all experiments, a drug-free growth control is required to separate the effect of the intervention from the natural bacterial growth characteristics. The drug-free growth control should be fed with the same growth medium (and supplements or drug cofactors) used in the experimental arms. As is the norm for biological experiments, the drug-free growth control is ideally run simultaneously with the experimental arms and not sequentially. If an experiment with many experimental arms must be conducted consecutively, a drug-free growth control should be ideally included in each round. However, given the complexity of HFS experiments, it may be acceptable to use fewer drug-free growth controls, having first demonstrated the inter-experiment variability of the control experiment.

## Considerations for outcome measures in HFS experiments

### Bacterial sampling

#### Bacterial sampling from the cartridge

Safety measures should be taken following local biosafety guidelines. For BSL-2 and BSL-3 pathogens, bacterial sampling should be performed in a Class I (or higher) microbiological safety cabinet. To ensure staff safety, the cartridge should be moved from its mount into the cabinet while keeping the inflow, outflow, and drug tubing connected. However, for BSL-1 organisms, the cartridge could remain in the incubator to avoid large temperature fluctuations, if local biosafety guidelines allow it. The high-speed peristaltic pumps between the central reservoir and the cartridge should be switched off during bacterial sampling. To minimize contamination, the HFS operator should work aseptically, and Luer-lock ports should be used for sampling. Prior to sampling, the contents of the hollow fiber cartridge should be mixed by pushing and pulling the contents of the cartridge using two syringes connected to the sampling ports, and sampling should be done through the same port throughout the experiment. The volume withdrawn from the cartridge should be kept to a minimum to ensure that the total bacterial population is not significantly reduced. The total of the sample taken should not exceed 10% of the ECS volume per day. A cartridge with a larger volume is recommended when larger samples are needed. Although the total HFS volume decreases after sampling, the removed volume should not be replaced with fresh growth medium. The system re-establishes equilibrium via osmosis across the cartridge fibers, and replacement would cause a sudden drop in antibiotic concentration.

#### Sampling schedule

Depending on the research question to be answered, existing data can be used to design an HFS experiment. There are large differences in the growth rates of bacteria (e.g., Enterobacteriaceae vs. mycobacteria). The growth rate affects when PD samples need to be taken because they changes the rate at which the experimenter expects the drug to act on the bacterium.^18^^,^^19^ In addition, the minimum inhibitory concentration (MIC) of a bacterial isolate can also be helpful in designing the overall drug exposure to be simulated in the HFS. There may also be published data on static time-kill assays against the microorganism of interest, or this can be done before performing the HFS experiment. This information can guide the selection of doses to be simulated in the HFS and the design of bacterial sampling schedule.

Before inoculating a hollow fiber cartridge, a sample should be collected to confirm the inoculum’s bacterial load. Depending on the bacterial life cycle, more or less frequent bacterial samples should be taken to enable full characterization of the time-kill curve. The total duration of the experiment depends on the microorganism of interest and the research question to be answered. To characterize the antibiotic effects on bacterial cell death and growth, shorter total durations can be used (e.g., ≤96 h for fast-growing bacteria, and ≤14 days for slow-growing bacteria). To characterize the development of antibiotic resistance, a longer experimental duration is advised (e.g., ≥96 h for fast-growing bacteria, and ≥14 days for slow-growing bacteria).

#### Sampling for contamination

When samples are quantified for bacterial load, an undiluted sample should be plated on a nutrient-rich, non-selective growth medium, such as a 5% Columbia sheep blood agar plate, to monitor potential contaminations. This sample should be incubated under equivalent incubating circumstances as the experiment (e.g., temperature and CO_2_ percentage). Contamination tests are also advised before starting any experiment.

### PK outcomes

During an experiment, antibiotic concentrations in the ECS should be measured to confirm that the intended PK targets (C_max_, T_max_, and T_1/2_) have been accurately simulated at the site of bacterial growth, especially if there is a systemic equilibrium delay between the central reservoir and the ECS. To avoid depleting the ECS volume and disturbing bacterial density, ECS concentrations should be measured using the residual volume obtained during routine bacterial sampling. Where direct ECS sampling is not feasible, antibiotic concentrations in the central reservoir may be measured as a proxy to validate PK target attainment. Measuring the antibiotic concentration in very viscous samples, due to high bacterial densities, can be a challenge for some methods of antibiotic quantification. A validated method with high precision and accuracy, such as high-performance liquid chromatography-UV (HPLC-UV) or liquid chromatography-tandem mass spectrometry (LC-MS/MS),^20^ should preferably be used, although bioassays have also been reported.^21^ It is unnecessary to take a PK sample before the first dose, at T = 0, but it can be beneficial when using as a blank matrix for the PK assay. Afterward, it is important to take a sample at the estimated T_max_. Sufficient PK samples should be taken from the central reservoir and the ECS to measure drug concentrations and validate the correct simulation of the targeted PK profiles.

### PD outcomes

#### Replicates

Replicates include technical and biological repeats. Technical replicates are obtained when endpoints are measured more than once from the same sample and the same system, while biological replicas come from different HFS experiments with the same parameter settings. Following the CLSI M26-A guideline,^22^ every HFS experiment should contain at least 3 technical replicates (measures on the same sample) for each sample collected and 2 biological replicates (samples from different cartridges).^22^

#### PD bacterial readouts and detection methods

Quantification of bacterial loads of samples collected from HFS experiments may be carried out using various quantification methods (see Table 1), including: CFU enumeration on agar plates (gold standard), broth microdilution methods, optical density measurements, quantification of the most probable number (MPN), which estimates the viable number of bacteria in a sample, including non-culturable cells,^23^ or the use of the mycobacteria growth indicator tube (MGIT) system to quantify the time-to-positivity (TTP), an outgrowth readout. For all cases, the limit of bacterial quantification should be defined, measured, and reported. Providing a deeper overview of bacterial readouts and detection methods is outside the scope of the current document.

**Table 1:** Recommendations for suggested reporting of specifications in the HFS experiments, adapted from Sadouki et al.^9^

Section	HFS feature	Further explanation
Descriptive specifications	primary study aim	main research question of the HFS study
	microbial species	microbial species and strain inoculated into the bioreactor cartridge
	antimicrobial(s)	antimicrobial(s) administered to the HFS, solvent, manufacturer, and CAS number
	duration	duration of the HFS experiment in days
Technical specifications	mimicked dose^a^	dose being mimicked in the HFS in mg/L
	C_max_	peak concentration of antimicrobial(s) in mg/L
	C_max, ss_	peak concentration of antimicrobial(s) reached at steady state, in mg/L
	T_½_^b^	elimination half-life
	T_max_	time taken to reach C_max_, in h
	C_min_^a^	lowest concentration of the drug in a dosing interval
	*T*	time between antimicrobial dose administrations
	cartridge source	manufacturer of the cartridge used and catalog number
	fiber type	cartridge fiber type (e.g., cellulosic, polysulfone, or polyvinylidene fluoride)
	flow rate	rate between the central reservoir and the bioreactor cartridge and rate from the diluent reservoir to the central reservoir
	pump model	pump models used
	tubing^a^	bore size, length, and material of tubing used
	administration	route and details of administration to be mimicked, e.g., bolus or infusion, with details of rate and volume and where drug is added to the system
	*in vivo* conditions	site of infection considered, accounting for protein binding
	system volume	total volume of the HFS
	PK and PD sampling schedule	PK and PD sampling time points, and the ports from which the samples are taken
	drug quantification	method to quantify PK samples (e.g., LC-MS/MS)
Microbiological specifications	medium	name and manufacturer of the medium used in the HFS
	growth conditions	incubation temperature and pH during the experiment
	control	details of control experiment, i.e., drug-free arm
	sterility check	sterility check in the cartridge and the central reservoir
	inoculum	method used to quantify the bacterial load of the inoculum and PD samples (e.g., microdilution method), method of inoculum preparation, and stage of culture used
	CFU	CFU/mL of the microorganism quantified from the cartridge sampling (including T = 0)
	viability^a^	cell viability markers beyond CFU enumeration (e.g., flow cytometry)
	resistance^a^	resistance of microbial sample phenotypically quantified
	genotyping^a^	molecular testing of the microbial sample
	biological replicates	number of replicates of HFS experiments with identical settings
	technical replicates	number of repeat testing of endpoint measures from the same HFS experiment, e.g., number of CFU determinations
Infected macrophage model	cell line	cell line name and number
	multiplicity of Infection	the ratio (MOI) of bacteria:cells when inoculating
	incubation conditions^a^	incubation temperature and CO_2_ percentage
	infection method	how the cell line is infected
	infection duration	duration infection method
	cell densities	enumeration of cell densities

a Additional parameter not required for reporting.

b In case of multiphasic pharmacokinetics, specify the corresponding half-lives for each phase and time when the next phase starts.

#### PK/PD modeling

PK/PD modelling is a quantitative approach used to characterize the relationship between drug exposure and its effects on pathogens. Data generated from HFS can be applied to PK/PD models to simulate and predict antimicrobial activity under clinically relevant conditions. However, a detailed discussion of PK/PD modelling is beyond the scope of the current guideline, as such analyses may not be directly relevant to every experimental question addressed using the HFS-I. For readers interested in this topic, we recommend the comprehensive overview by Nielson et al.^24^ for further reading.

#### Further testing

Besides quantification of the number of viable bacteria in the hollow fiber cartridge, it might be of interest to perform other tests including, but not limited to, targeted investigations of the emergence of antibiotic resistance and the genome, transcriptome, or the various omics. These applications are specially indicated for HFS experiments due to the high bacterial cell densities and long incubation times that can be achieved within the system. Some of these methods may demand too large a sample from the hollow fiber cartridge, and this may impact the total bacterial population; as indicated above, a maximum sampling of 10% of the cartridge volume per day is advised. Larger cartridge types might be an option in case a larger volume is required. Providing an overview of methods as well as reflecting on these methods are outside the scope of the current document.

## Waste management

### Biological hazard

The way the hollow fiber cartridges are discarded depends on the biological hazard of the pathogen that is being studied. When using BSL-2 organisms, the drug and the inflow and outflow tubing may be disconnected, leaving an enclosed circuit consisting only of the central reservoir and the hollow fiber cartridge. The hollow fiber cartridge may be directly flushed with a disinfectant (e.g., bleach) and left for appropriate contact time, following local guidelines. Afterward, the side ports of the cartridge may be closed, and the cartridge may be disconnected from the central reservoir and handled as BSL-2 waste (follow local guidelines and in-house procedures when in doubt).

In case BSL-3 pathogens are being used, all pumps should be stopped, and side ports of the hollow fiber cartridge should be closed. A syringe should be filled with relevant phenolic-based disinfectants, and the cartridge should be evacuated, while simultaneously using another syringe to evacuate the extracapillary fluid. The ECS fluid should be incubated overnight with sterilizing agents. The disinfectant-filled hollow fiber cartridge should be maintained overnight to ensure adequate exposure, before removing it from the laboratory, according to local regulations that include autoclaving for disposal.

### Reuse of materials

Tubing, Luer-lock connectors, caps, diluent and elimination reservoirs, central reservoir, and filters (dependent on the material) can be reused. Most of these materials can be autoclaved multiple times, and their performance is not affected when used in a new experiment. For other materials (tubing and filters), however, the investigator should be aware that the performance tends to decrease with repeated use. Reusing tubing can lead to leaks and microcracks in the regions subjected to compressive stress, which, in turn, increases the risk of contamination and biohazard. Also, the part that enters the peristaltic pump flattens out over time, changing the set flow rate and increasing the risk of damage. This part should be replaced regularly, although the rest of the tubing can be reused. As the filters are used several times, they may fail and their function may deteriorate.

## Reporting standards

The detail in the reporting of experimental parameters varies between publications and, consequently, data cannot be validated or reproduced in all cases.^9^ To ensure robust reproducibility, we propose that HFS experiment reports must contain a minimum set of details of the experimentally confirmed and target parameters as described in Table 1.

### Workplan

**Figure 5:**
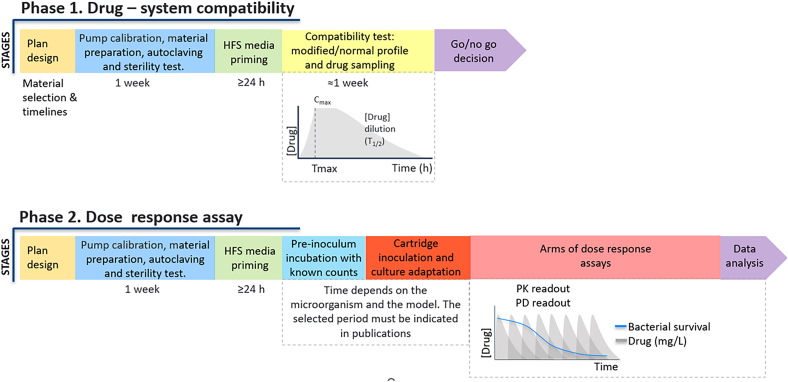
Stages of an HFS experiment

It is advisable to draw up a workplan that includes a detailed time table for the experiment. Figure 5 shows an overview of the different phases of an HFS experiment. Examples of auxiliary schedules can be found in Tables 2 and 3. In addition, a pre-experiment checklist can be used to check whether sufficient preparatory steps have been carried out before starting the experiment (Table 4).

**Table 2:** List of equipment necessary for HFS experiments

Item	Example picture
Low- and high-speed pumps	
The low-speed pumps are used to pump fresh medium both into and out of the central reservoir to simulate half-life of the antibiotic tested in the system. The high-speed pump is used to pump the medium from the central reservoir into the hollow fiber cartridge and back.	
Syringe driver	
The syringe driver is used to accommodate the syringe (usually 20 mL or 50 mL) with the antibiotic solution and to control the infusion of the drug into the HFS.	
Tubing	
(Platinum cured) silicone tubing used in the HFS connects the diluent reservoir to the central reservoir and the central reservoir to the elimination reservoir. It can also be used to connect the central reservoir to the hollow fiber cartridge.	
Filter unit	
The filter units are located on the central reservoir, the elimination reservoir, and the diluent reservoir. They are used to absorb pressure fluctuations due to changes in the liquid in these containers. A filter can also be inserted between the 3-way stopcock and the clave connector for taking pharmacokinetic samples so that nothing can get into the central reservoir.	
Culture guard	
A culture guard can be connected to the reservoir cap of the diluent reservoir and to the tubing leading to the central reservoir. This filter is a small hollow fiber filter with a large surface area that filters all the growth medium that enters the central reservoir. If the diluent reservoir is contaminated, the central reservoir remains protected.	
Clave connector	
Clave connectors open when a Luer-lock syringe is connected. They are used as a connector to which syringes can be attached to take pharmacokinetic samples and the samples of bacterial cultures from the cartridge.	
Central reservoir (100–450 mL)	
The central reservoir is the center piece of the HFS. In the central reservoir, the desired pharmacokinetic curves are achieved by pumping in the diluents and drug, while pumping out an excess of liquid. The contents of the central reservoir are pumped into and out of the hollow fiber cartridge.	
3-way stopcock	
Three-way stopcocks can be inserted between the central reservoir and the tube coming out of the hollow fiber cartridge. The third port can then be connected to the inflow tubing (if it is not directly connected to the central reservoir) and to the drug inflow tubing coming out of the syringe driver.	
Hollow fiber cartridges	
Hollow fiber cartridges are closed cylinders packed with semi-permeable fibers. They form a closed loop system when connected to a central reservoir with inlet and outlet pipes. A cartridge has 4 ports, 2 of which are connected to the central reservoir and the other 2 are used to connect syringes for sampling bacterial cultures.	
Central reservoir caps Diluent reservoir caps Elimination reservoir caps	
The caps for the central reservoir (>4 openings), the diluent, and the elimination reservoir (2 openings) are placed on their corresponding bottles. They are used to close the reservoirs tightly and to connect the individual reservoirs.	
Luer-lock fitting male Luer-lock fitting female	
Luer-lock fittings are used at both ends of tubes to connect them firmly to other parts of the system.	
Diluent reservoir (>1 L) Elimination reservoir (>1 L)	
The diluent and elimination reservoirs serve to store growth medium, which is used to dilute the growth medium in the central reservoir and to store the waste coming from the central reservoir.

**Table 3:** Overview of bacterial readout methods

Quantification method	Feature	Further explanation
CFU on whole plates per dilution	method	sample is 10-fold serially diluted; from each dilution, a large volume (≥100 μL) is plated on an individual plate; after incubation, colonies are counted.
	advantages	gold standard provides an accurate determination of bacterial density can have a very low limit of detection
	disadvantages	bacteria that form clumps decrease the estimated bacterial density. can be very laborious if multiple technical replicates are included.
Broth microdilution	method	sample is 10-fold serially diluted; three drops of 10 μL (or one drop of 20 μL) from each dilution are plated on a plate; after incubation, colonies are counted.
	advantages	easy to perform provides an accurate determination of bacterial density technical replicates can be easily added
	disadvantage	lowest limit of detection is usually around 1-log_10_ CFU/mL
Optical density	method	using a densitometer, the optical density at 600 nm of the sample is measured; the measured OD_600_ correlates with the bacterial density.
	advantages	very easy to perform quick results cheap
	disadvantages	optical and bacterial density must correlate well provides only an estimation of the bacterial density
MPN	method	multiple tubes are filled with growth medium and are inoculated with 10-fold differences of inoculum volume (e.g., 15 glass tubes with 3 different inoculum sizes), which are then incubated; depending on the number of tubes containing growth, medium this is compared to an MPN that provides the corresponding MPN in cells/mL.
	advantages	easy to perform and compatible with a wide range of samples.
	disadvantages	does not provide an absolute cell density can be laborious.
MGIT TTP	method	in an MGIT tube, oxygen inhibiting a fluorophore is replaced with carbon dioxide when bacteria grow; this replacement activates the said fluorophore; the time required for this activation—time-to-positivity (TTP)—is a measure of bacterial density.
	advantages	continuous measurement easy to inoculate objective readout has low limits of detection
	disadvantages	can be difficult to interpret when bacteria are under antibiotic stress expensive

**Table 4:** Pre-HFS checklist

Pre-HFS checklist	
1.	Does your research question need the HFS?
2.	Is your microorganism well characterized and available for distribution to other labs?
3.	Do you have MIC data of study drugs, from gold standard/guideline-compliant methods in relevant media?
4.	Do you have PD assessments of your drug-bacterium combination from time-kill experiments?
5	Do you have literature or proprietary information available on the PK profile you want to mimic in the HFS?
6.	Have you done a growth curve of your target microorganism in the HFS in the relevant (supplemented) media and type of cartridge?
7.	Do you achieve the desired PK in the ECS (i.e., correct pump settings and correct cartridge type for the study drug)?
8.	Do you have all supplies (media, drugs, plates, and tubing) in stock?
9	Do you have a validated analytical method (e.g., HPLC-UV or LC-MS) to quantify your drug of interest in the media used in the HFS?

**Extra checks for infected macrophage models**	

10.	Have you done a growth curve of your cells in the HFS in the relevant media?
11.	Have you done preliminary coinfection experiments with cells and bacteria?
12.	Do antibiotics of choice harm the cells?

## Future directions

The use of pre-clinical PK/PD sciences is currently well embedded in the development of clinical antibiotic regimes. Within HFS experiments, the relevance of hollow fiber cartridge selection is being increasingly recognized, although the quantitative impact of individual cartridge properties on drug exposure, pathogen behavior, and PD readouts in pre-clinical experiments remains poorly characterized. Systematic studies disentangling the effects of membrane chemistry, separation characteristics, cartridge geometry, and physical design on antibiotic distribution and behavior are currently lacking, yet such factors may critically influence experimental outcomes and contribute to inter-study variability. Furthermore, the implementation of an in-line oxygen sensor in the HFS-I has not yet been reported, and although it may be of interest, its impact has not been systematically evaluated. Addressing these knowledge gaps through standardized reporting, comparative cartridge evaluations, and dedicated methodological investigations may substantially improve access to this specialized methodology. The use of the standardized method described in the current report initiates the possibility to address these knowledge gaps.

## Acknowledgments

We would like to acknowledge valuable expert review, recommendations, and endorsement from the European Society of Clinical Microbiology and Infectious Diseases PK/PD of Anti-Infectives Study Group (ESCMID EPASG). This work has been partially supported by the Innovative Medicines Initiatives 2 Joint Undertaking (grant no. 853989) to S.-R.G. and A.L. The Joint Undertaking (JU) receives support from the European Union’s Horizon 2020 Research and Innovation Programme, 10.13039/100013322EFPIA, Global Alliance for TB Drug Development Non-Profit Organization, and 10.13039/100000865Bill & Melinda Gates Foundation, 10.13039/100008890University of Dundee (http://www.imi.europa.eu.) This work reflects only the author’s views, and the JU is not responsible for any use that may be made of the information it contains. F.K. is the recipient of Sir Henry Dale Fellowship jointly funded by the 10.13039/100010269Wellcome Trust and the 10.13039/501100000288Royal Society (grant no. 220587/Z/20/Z).

## Declaration of interests

The authors declare no competing interests.

## Figures and Tables

**Table 5:** Overview of pharmacokinetic formulas that can be used to define drug inflow

Parameter	Symbol	Description	Formula
Clearance (mL/min)	CL	the rate at which the drug is being eliminated from the system and correlates with the speed of the low-speed peristaltic pumps in mL/min.	$CL=\frac{Vd\cdot \ln\left(2\right)}{T_{1/2}}$ *CL* = *Vd*·*k*_*e*_
Elimination rate constant (h^−1^)	K_e_	the rate at which the drug is eliminated from the system.	$k_{e}=\frac{CL}{Vd}$
Concentration (mg/L)	C	concentration at time (*t*)	$C=\frac{K_{0}}{CL}\cdot \left(1-e^{-k_{e}\cdot t}\right)$ *C*=*C*_0_·*e*^-*ke*·*t*^
Infusion rate (mg/h)	K_0_	the rate at which the antibiotic is infused.	$K_{0}=\frac{CL\cdot C_{\max}}{1-e^{-ke\cdot t}}$
Half-life (h)	T_1/2_	the time it takes for the concentration of the drug in the system to be reduced by 50%.	$T_{1/2}=\frac{\ln\left(2\right)}{k_{e}}$
Where:			
Volume of distribution (L)	V_d_	the total volume of the hollow fiber cartridge, closed loop to the central reservoir, and the central reservoir	–
Time since dose (h)	t	the time since the last dose in h	–
Maximum concentration (mg/L)	C_max_	the maximum concentration reached in the system	–

**Table 6:** Overview of potential problems, causes, and solutions when performing HFS experiments

Problem	Potential cause(s)	Solution(s)
Contamination	inadequate aseptic technique during setup or sampling	use sterile technique and work in a biosafety cabinet
	leaky connections	tighten or replace fittings and tubing
Flow rate instability	pump malfunction	calibrate/maintain pump before use
	tubing obstruction	flush and replace obstructed tubing
	airlocks in the system	remove air bubbles and restart
Air bubble formation	incomplete system priming	fully prime system with sterile medium
	leaky connections	check and secure all connections
	accumulation due to oxygenation tube	manually extract air bubbles
Clogging of hollow fiber cartridges	precipitation of drug or protein build-up	filter and pre-dissolve drugs
	high bacterial burden	reduce inoculum or passage frequently
	clumping of bacteria	use detergents
Unexpected drug loss	drug binding to cartridge/membranes	assess drug binding to HFS components
	adsorption to HFS components	switch to low-binding materials or a pre-equilibrate system or develop a saturation strategy
Poor bacterial growth	incorrect media composition	optimize media formulation
	suboptimal temperature or pH	use temperature and pH controls
Backflow or cross-contamination	improper placement of waste/output tubing	ensure correct tubing orientation and gravity flow
	pressure imbalance	balance pressure using vented caps
Inconsistent sampling volumes	manual pipetting error	use calibrated pipettes or peristaltic sampling
	sample port clogging	clean or replace sample ports
Hollow fiber cartridge membrane rupture or leakage	overpressure	monitor pressure limits
	manufacturing defect	inspect cartridges before use
	rough handling	handle with care
pH drift over time	CO_2_ accumulation	use buffered medium (e.g., HEPES)
	inadequate buffering	ensure proper gas exchange in the system

## Appendices

### Appendix A: Cartridge selection

The suitability of the HFS cartridge is a critical determinant of experimental validity in this model. The validation of the antibiotic-cartridge compatibility and its experimental protocol are described in validation of antibiotic profiles in the system. Furthermore, the choice of an HFS cartridge should be guided by integrating data from the physicochemical properties of the antimicrobial agent, biological characteristics of the pathogen, and the intended PK simulation. Commercially available cartridges differ in membrane material, hydrophilicity, nominal molecular weight cut-off (MWCO) or pore size, geometry, and mechanical properties, all of which may influence drug exposure and pathogen containment. Here, we describe rule-of-thumbs to assist in choosing suitable cartridges.

In general, both hydrophilic and hydrophobic antibiotics (e.g., compounds with low and high logP values,^25^ respectively) should be tested using cartridges equipped with hydrophilic membranes to minimize non-specific adsorption to the membrane pore walls. In fact, hydrophobic membranes might not let hydrophilic antibiotics cross the membrane,^26^ and the adsorption of lipophilic antimicrobials to polymeric materials may lead to systematically reduced effective drug concentrations within the ECS. In contrast, hydrophilic compounds are generally less prone to membrane adsorption, although pre-experimental drug recovery testing remains advisable, as described in validation of antibiotic profiles in the system of the main text.

Membrane separation characteristics must be selected to ensure reliable pathogen retention while permitting diffusion of the unbound drug fraction. For bacterial infection models, ultrafiltration membranes with MWCO values typically of ≤20 kDa are preferred, as larger-pore microfiltration membranes may permit pathogen leakage into the central reservoir, compromising sterility and pharmacodynamic (PD) interpretation. In addition, for antibiotics with substantial plasma protein binding,^27^ the MWCO must be sufficiently large to allow diffusion of free drug while ensuring retention of carrier proteins. If the protein molecular weight exceeds the membrane MWCO, protein-bound drug may be excluded from the ECS, leading to underestimation of clinically relevant exposure. However, this aspect would not be of relevance when the unbound free-drug fraction is mimicked using protein-free synthetic media.

Beyond membrane material and MWCO, cartridge geometry and physical design parameters warrant consideration. The effective membrane surface area-to-volume ratio influences the diffusion kinetics and equilibration time between the central reservoir and the ECS, with higher ratios generally reducing diffusion-limited delays. Fiber inner diameter and wall thickness determine diffusion distance and hydraulic resistance and may affect the temporal fidelity of rapidly changing pharmacokinetic (PK) profiles. Similarly, cartridge hold-up volume and dead volume within headers and tubing can distort simulated concentration-time profiles, particularly for drugs with short half-lives or during pulsed dosing regimens.

Additional cartridge properties may further influence experimental outcomes, especially in long-duration or pathogen-specific models. Although there is no clear evidence of the extent of the impact in antimicrobial PK/PD studies, membrane gas permeability of the oxygenation tube can affect oxygen availability within the ECS and may be relevant for obligate aerobes, slow-growing organisms, or biofilm-forming pathogens. The availability of oxygen might impact the bacterial growth dynamics. In-line oxygen sensors^28^ may be placed before and after the hollow fiber cartridge to measure oxygen concentration in the medium when using oxygen-sensitive bacteria. In this case, before selecting a cartridge, the permeability of oxygen through the cartridge membrane should be evaluated by referring to the literature, as not all membranes are oxygen permeable.^29^ Mechanical robustness and pressure tolerance of the HFS cartridge should be sufficient to maintain stable membrane properties throughout the experiment, as membrane deformation or micro-leakage may alter separation characteristics over time.^30^ Finally, sterilization compatibility, potential extractables or leachables from the cartridge, and batch-to-batch variability should be considered, as these factors may affect microbial growth, analytical measurements, or drug stability during prolonged exposure.

Collectively, these considerations highlight that the selection of hollow fiber cartridges should be based as far as possible on a structured assessment of membrane chemistry, separation properties, cartridge geometry, and mechanical characteristics. As there are no industry-wide standards, we recommend testing the experimental cartridge suitability for each antibiotic used following the description in validation of antibiotic profiles in the system to limit expenses and experimental suitability testing. Explicit reporting of these parameters may improve reproducibility and facilitate the comparison of HFS infection studies across laboratories.

### Appendix B: Considerations for drug-system compatibility

The physicochemical properties of the drug must be considered when selecting the cartridge fiber material. A compatibility test must be performed as other major components of the HFS are polypropylene, polystyrene, polycarbonate, and (platinum cured) silicone materials that may retain antibiotics. The test consists of infusing the drug into the system to mimic a desired PK profile and measuring the drug concentration in the ECS and central reservoir over time. This verifies whether the drug binds to the system but also ensures that the PK profile is successfully achieved with the selected pump settings. Figure 5 shows suggestions for compatibility tests to be performed.

A modified PK profile of the drug could be targeted in a media broth without bacteria (Figure 5A). This test targets the desired C_max_ at the expected T_max_ with a C_max_ holding step of ca. 90 min before dilution of the drug. The C_max_ holding step is achieved by recirculating the drug solution between the central reservoir and the cartridge without diluting the broth. This rapid test can be performed for one day or less if the T_1/2_ is shorter. It shows the lag time of drug concentration between the central reservoir and the ECS and the time it takes to reach equilibrium in the system. It also shows whether T_max_, C_max_, and T_1/2_ were properly reached at the first drug infusion.

The simulation of the “targeted profile” (Figure 5B) is recommended to imitate more than two drug infusions, depending on what further experiments are required. The dosing aims either to increase C_max_ until steady state is reached or to reach C_max,ss_ directly. This test is designed to show whether C_max_ is achieved correctly over multiple dose infusions or whether the experiment needs drug balancing at certain points. In addition, this test will ensure that the system is using the correct pump settings for the desired PK parameters. Similarly, drug binding will be detected if target concentrations are not achieved, or/and if concentrations are different between the central reservoir and the ECS. It is recommended to target concentrations mainly in the ECS where the pathogens reside.

Compatibility tests are conducted prior to dose-response assays and do not replace further PK measurements in actual experiments.

### Appendix C: Pharmacokinetic formulas that can be used to define drug inflow

The formulas used in Table 5 are fully incorporated in freely available web applications HF-App^13^ or PKPDIA.^14^ For further reading and information, we would like to refer to studies by Aranza-Climent et al. (HF-APP)^13^ and Kloprogge et al. (PKPDIA).^31^

### Appendix D: List of the necessary equipment for HFS experiments and manufacturer suggestions

See Table 2.

### Appendix E: List of pharmacodynamic readouts

See Table 3.

### Appendix F: Troubleshooting table

See Table 6.

## References

[1] European Medicines Agency (2015).

[2] Belmatoug N., Fantin B. (1997). Contribution of animal models of infection for the evaluation of the activity of antimicrobial agents. Int. J. Antimicrob. Agents.

[3] Zak O., O'Reilly T. (1991). Animal models in the evaluation of antimicrobial agents. Antimicrob. Agents Chemother..

[4] Wale Y.M., Roberts J.A., Sime F.B. (2024). Dynamic In Vitro PK/PD Infection Models for the Development and Optimisation of Antimicrobial Regimens: A Narrative Review. Antibiotics.

[5] Cadwell J.J., Whitford W.G. (2017). Technology Platforms for 3D Cell Culture: A User's Guide.

[6] Noble M.A. (2020).

[7] Aguilar-Ayala D.A., Sanz-García F., Rabodoarivelo M.S., Susanto B.O., Bailo R., Eveque-Mourroux M.R., Willand N., Simonsson U.S.H., Ramón-García S., Lucía A., ERA4TB consortium (2024). Evaluation of critical parameters in the hollow-fibre system for tuberculosis: A case study of moxifloxacin. Br. J. Clin. Pharmacol..

[8] FiberCell Systems Inc. (2023). Fibercell Systems Duet Pump.

[9] Sadouki Z., McHugh T.D., Aarnoutse R., Ortiz Canseco J., Darlow C., Hope W., van Ingen J., Longshaw C., Manissero D., Mead A. (2021). Application of the hollow fibre infection model (HFIM) in antimicrobial development: a systematic review and recommendations of reporting. J. Antimicrob. Chemother..

[10] Sanz-García F., Ndjogou D., Aguilar-Ayala D.A., Eveque-Mourroux M.R., Benítez A., Aznar S., Isach-Traver N., Mendoza-Losana A., Willand N., Lucía A., Ramón-García S. (2026). An assay toolkit for anti-tuberculosis drugs in the hollow fiber system. iScience.

[11] Blaser J., Stone B.B., Zinner S.H. (1985). Two compartment kinetic model with multiple artificial capillary units. J. Antimicrob. Chemother..

[12] Readman J.B., Acman M., Hamawandi A., Chiu C.H., Sharland M., Lindsay J.A., Standing J.F. (2023). Cefotaxime/sulbactam plus gentamicin as a potential carbapenem- and amikacin-sparing first-line combination for neonatal sepsis in high ESBL prevalence settings. J. Antimicrob. Chemother..

[13] Vincent Aranzana-Climent, A.C., Nicolas G. (2021). HF-App: A R-Shiny application to streamline hollow-fibre experiments. R application version 1.0.0.

[14] PKPDIA HFS App. https://pkpdia.shinyapps.io/hfs_app/.

[15] (2019).

[16] Brook I. (1989). Inoculum effect. Rev. Infect. Dis..

[17] Clinical & Laboratory Standards Institute (2023).

[18] Rodríguez-Ochoa J.L., Pérez-Palacios P., Merino-Bohórquez V., Ortiz-Padilla M., Velázquez-Escudero A., Rodríguez-Baño J., Rodríguez-Martínez J.M., Pascual Á., Docobo-Pérez F. (2024). Evaluation of temocillin efficacy against KPC-2-producing Klebsiella pneumoniae isolates in a hollow-fibre infection model. J. Antimicrob. Chemother..

[19] Kloprogge F., Ortiz Canseco J., Phee L., Sadouki Z., Kipper K., Witney A.A., Stoker N., McHugh T.D. (2022). Emergence of phenotypic and genotypic antimicrobial resistance in Mycobacterium tuberculosis. Sci. Rep..

[20] European Medicines Agency (2022).

[21] Dafale N.A., Semwal U.P., Agarwal P.K., Sharma P., Singh G.N. (2015). Development and validation of microbial bioassay for quantification of Levofloxacin in pharmaceutical preparations. J. Pharm. Anal..

[22] Clinical & Laboratory Standards Institute (1999).

[23] Rabodoarivelo M.S., Hoffmann E., Gaudin C., Aguilar-Ayala D.A., Galizia J., Sonnenkalb L., Dal Molin M., Cioetto-Mazzabò L., Degiacomi G., Recchia D. (2025). Protocol to quantify bacterial burden in time-kill assays using colony-forming units and most probable number readouts for Mycobacterium tuberculosis. STAR Protoc..

[24] Nielsen E.I., Friberg L.E. (2013). Pharmacokinetic-pharmacodynamic modeling of antibacterial drugs. Pharmacol. Rev..

[25] Leo A.J. (1991). Methods in enzymology.

[26] Jiang S., Hu T., Xiong Z., Yang Z., Wu T., Zhou H., Zhang J. (2025). Rejection of antibiotics using modified polyvinylidene fluoride (PVDF) membranes with polyethyleneimine co-blending: Relating membrane performances to operating conditions. Desalination Water Treat..

[27] Beer J., Wagner C.C., Zeitlinger M. (2009). Protein binding of antimicrobials: methods for quantification and for investigation of its impact on bacterial killing. AAPS J..

[28] Rivera K.R., Yokus M.A., Erb P.D., Pozdin V.A., Daniele M. (2019). Measuring and regulating oxygen levels in microphysiological systems: Design, material, and sensor considerations. Analyst.

[29] Hazarika G., Ingole P.G. (2024). Polymer-based hollow fiber membranes: A modern trend in gas separation technologies. Mater. Today Chem..

[30] Cytiva (2022).

[31] Kloprogge F., Hammond R., Kipper K., Gillespie S.H., Della Pasqua O. (2019). Mimicking in-vivo exposures to drug combinations in-vitro: anti-tuberculosis drugs in lung lesions and the hollow fiber model of infection. Sci. Rep..

